# Minority Populations and Health: An Introduction to Health Disparities in the United States

**Published:** 2005-09-15

**Authors:** Sandra Headen

**Affiliations:** Research, Program Implementation and Evaluation, The Paragon Foundation, Raleigh, NC

**Figure:**
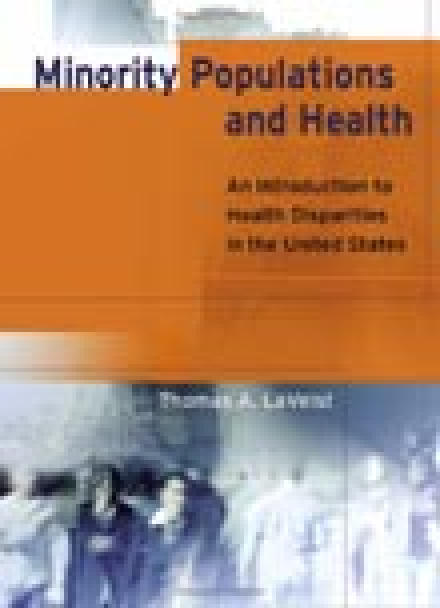


*Minority Populations and Health: An Introduction to Health Disparities in the United States*, by Thomas A. LaVeist, provides a theoretical and methodological framework for examining the determinants of health and factors that contribute to health disparities among racial and ethnic groups in the United States. Following an excellent analysis of the magnitude of the health disparities problem, the author presents conceptual models for developing policies and interventions that can reduce disparities and improve health outcomes and services for all groups.

This textbook is more comprehensive than many works on minority health, offering instruction on a range of topics required to understand the literature on health disparities. Although aimed at undergraduates, it is so thorough, well written, and elegant in design that it should be required reading for graduate students, practicing health professionals — including physicians — and anyone seeking a deeper understanding of how health disparities have developed and are maintained.

The text is state-of-the-art in its analysis of health disparities from both domestic and international perspectives. Health indicators for the five racial/ethnic groups in the United States are compared with populations in 13 developing and developed countries around the world. One example (Figure 4.8) shows the startling observation that African Americans had a higher infant mortality rate in 1999 than other racial/ethnic groups in the United States and higher than populations in 12 other countries, including some developing nations. Russia was the only country whose infant mortality rates exceeded those of African Americans.

When possible, data for subgroups within racial or ethnic categories are presented separately in the book, further illuminating the scope of the disparities problem. In an example from 2000 census data (Figure 4.7), infant mortality rates for the five major racial/ethnic groups are compared with subgroups of Asians/Pacific Islanders (Chinese, Japanese, Filipino, Hawaiian, Other) and Hispanics/Latinos (Mexican, Puerto Rican, Cuban, Central/South American, Other). These comparisons reveal significant within-group health disparities in addition to disparities between groups. Among Asians, Hawaiians had the highest infant mortality rates, and among Hispanics, Puerto Ricans had the highest rates.

The book is divided into five sections composed of 14 chapters and two appendices. An introductory chapter describes the historical roots of inequality in America — the enslavement of African Americans and the decimation of American Indians through war, disease, and displacement. Part One, "Crosscutting Issues," includes two chapters. The first addresses conceptual problems with the categories used to measure health disparities and the author's discomfort with them. The second introduces the tools of demography to describe the distribution of racial and ethnic groups in the United States and the role of fertility, mortality, and migration in population growth. Part Two, "Morbidity, Mortality, and Racial/Ethnic Disparities in Health," includes chapters on the epidemiology of physical health conditions, mental health, and health care services. Part Three, "Etiology of Racial/Ethnic Differences in Health," covers three topics: theories that explain racial and ethnic group differences, explorations of the role of socioeconomic status in racial and ethnic differences, and theories of and empirical research on behavior and health.

Part Four, "Racial/Ethnic Group-Specific Health Issues," includes four chapters that underscore the value of this book as a primary text and as a reference for a comprehensive review of health disparities. These chapters are also excellent examples of the conceptual coherence of the book. Each begins with an outline of the topics that will be covered: health status (death rates and causes of death), health care access and use, and major health risks and health issues unique to each group. A chapter summary and list of key words are also provided. To avoid density and wordiness, some definitions and related facts are presented as sidebars to the text. The selection and interpretation of data in the text of each chapter builds skills in understanding health statistics and makes the book delightful and informative to read.

Part Five, "Conclusions," includes a synthesizing chapter and two appendices which supplement material in the text with additional reading and case studies that highlight health-related problems that members of specific racial/ethnic groups might encounter. As would be expected from a scholar who teaches and publishes in the field, LaVeist offers several conceptual models in chapter 14 that present bold solutions to reduce health disparities. One is an action-oriented blueprint that identifies points of intervention which have been demonstrated to affect health outcomes. These interventions target socioenvironmental factors, such as eliminating exposure to environmental hazards; individual-level factors, such as improving English proficiency and increasing health screening; and factors related to the health care system, such as providing universal access to health care and pharmaceuticals. Finally, the author suggests that the proposed solutions should be tested empirically in controlled research studies that are community-based, participatory, and tailored to the needs of racial and ethnic minority groups.

*Minority Populations and Health: An Introduction to Health Disparities in the United States* is a welcome addition to the field because it widens access to the complex issues underlying the health disparities problem. With greater knowledge and understanding, health professionals can more enthusiastically embrace the paradigm shift that redirects the focus of health and health care away from the white majority and toward the diverse experiences of racial and ethnic minorities.

